# Nitrogen-Doped Carbon Dots Induced Enhancement in CO_2_ Sensing Response From ZnO–Porous Silicon Hybrid Structure

**DOI:** 10.3389/fchem.2020.00291

**Published:** 2020-05-05

**Authors:** Jesús A. Ramos-Ramón, Naveen K. R. Bogireddy, Jorge Arturo Giles Vieyra, Tangirala V. K. Karthik, Vivechana Agarwal

**Affiliations:** ^1^Centro de Investigación en Ingeniería y Ciencias Aplicadas, Universidad Autónoma del Estado de Morelos, Cuernavaca, Mexico; ^2^Departamento de Metal-Mecánica, Instituto Tecnológico de Zacatepec, Instituto Nacional de México, Zacatepec de Hidalgo, Mexico; ^3^Ingeniería Industrial, Universidad Autónoma del Estado de Hidalgo, Pachuca, Mexico

**Keywords:** zinc oxide, carbon quantum dots, porous silicon, gas sensing, luminescence

## Abstract

In this study, we report a simple method for the fabrication of carbon dots sensitized zinc oxide–porous silicon (ZnO–pSi) hybrid structures for carbon dioxide (CO_2_) sensing. A micro-/nanostructured layer of ZnO is formed over electrochemically prepared pSi substrates using a simple chemical precipitation method. The hybrid structure was structurally and optically characterized using scanning electron microscopy, X-ray diffraction, fluorescence, and cathodoluminescence after the incorporation of hydrothermally prepared nitrogen-doped carbon dots (NCDs) by drop casting. With respect to the control sample, although all the devices show an enhancement in the sensing response in the presence of NCDs, the optimal concentration shows an increase of ~37% at an operating temperature of 200°C and a response time <30 s. The increment in the CO_2_-sensing response, upon the addition of NCDs, is attributed to an increase in CO_2_-oxygen species reactions on the ZnO surface due to an increment in the free electron density at the metal–semiconductor-type junction of NCD clusters and ZnO micro-/nanorods. A significant increase in the sensing response (~24%) at low operating temperature (100°C) opens the possibility of developing very large-scale integrable (VLSI), low operational cost gas sensors with easy fabrication methods and low-cost materials.

**Figure:**
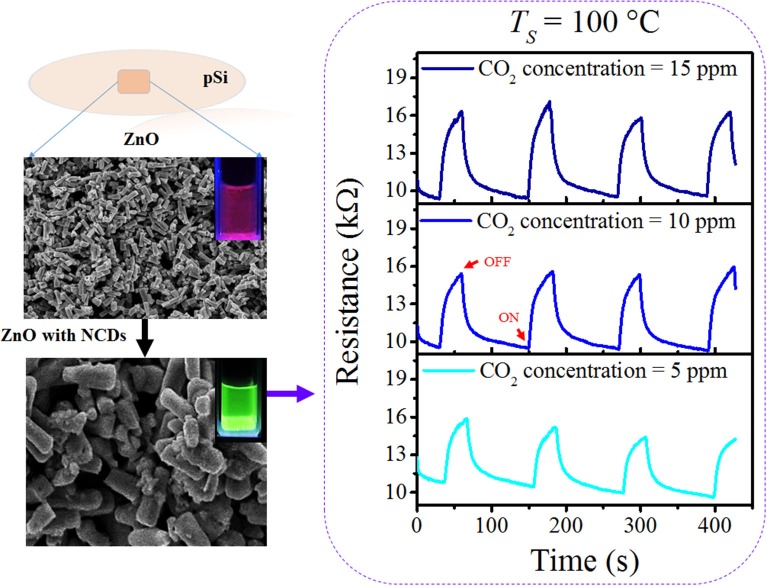
GRAPHICAL ABSTRACT

## Introduction

Chemical sensors have been in high demand due to their application in various fields such as environmental pollution monitoring and control, healthcare, food industries, etc. (Choi et al., 2019). Particularly, the solid-state chemical sensors are highlighted due to their compatibility with microelectronic processing, high thermal and chemical stability, and relatively low-dimension manufacturing for the sensing of different sorts of gases such as H_2_S, CO, O_3_, and so on (Yamaura et al., 1996; Suchea et al., 2006; Cuong et al., 2010; Song et al., 2016). However, the relatively high production costs represent an obstacle for their implementation. Utilizing simple fabrication methods and low-cost materials is a major challenge for reducing costs in their mass production. Carbon/graphene dots (CDs) have received particular interest in the last years due to their low production cost, high abundance, low environmental impact, and physical properties, and numerous applications, such as solar cells' electrodes (Rodríguez-Pérez et al., 2017), for the imaging of human cells (Wang and Zhou, 2014), up- and down-conversion effect (Zhuo et al., 2012; Lim et al., 2015), and so on. CDs can be fabricated by the bottom–up or the top–down approaches from different carbon sources, such as sucrose (Zhang et al., 2010), candle soot (Liu et al., 2007), graphite (Li et al., 2010), milk (Wang and Zhou, 2014), or citric acid (Song et al., 2015; Simões et al., 2016; Bogireddy et al., 2019). However, in some cases, the preparation methods require specific setups or highly controlled reaction conditions, which increase the fabrication costs and time. Different carbon structures [such as dots, single-/multiwalled nanotubes, graphene/reduced graphene oxide (RGO) sheets] have been mixed with different metal oxides for photodegradation of pollutants (Daneshvar et al., 2004; Guo et al., 2009; Li et al., 2013; Trinh et al., 2016; Vinayagam et al., 2018) and as gas sensors (Cuong et al., 2010; Su and Pan, 2011; Yin et al., 2014; Song et al., 2016; Chen et al., 2017; Schütt et al., 2017). For instance, Song et al. (2016) developed highly sensitive SnO_2_ quantum wires anchored on RGO nanocomposite sensor for the detection of H_2_S gas operating at room temperature, reducing the operating power consumption. Yin et al. fabricated SnO_2_ nanoparticles on RGO sheet composites through rapid microwave-assisted synthesis for the detection of H_2_S gas, demonstrating a fast response at 100°C of operating temperature and low gas concentration (Yin et al., 2014). Cuong et al. prepared an aligned ZnO nanowire array on graphene for the detection of H_2_S in oxygen at room temperature (Cuong et al., 2010). The fabricated sensor was able to detect H_2_S in low concentration (down to 2 ppm) due to the release of oxygen molecules during thermal reduction and evaporation of the by-product process. Those oxygen molecules are adsorbed on the surface of ZnO nanorods (NRs) improving gas sensitivity.

In addition, all the semiconductor chemical sensors are based on adsorption and desorption of target gas molecules on the metal oxide surface resulting in a change in surface electrical resistance. The interaction of metal oxide such as ZnO (*n*-type semiconductor), with oxidizing gases like CO_2_, results in an increase in the surface resistance, whereas for reducing gases like CO or propane, the surface resistance of ZnO decreases. In addition, CO_2_ molecule can be physisorbed at room temperature in linear and parallel configurations and chemisorbed in the form of bent species over the metal oxide at temperatures between 100 and 300°C (Burghaus, 2014). The linear, parallel, and bent species configuration of CO_2_ is over metal sites of metal oxide, and CO_2_ generally form carbonates [(CO_3_)^2−^] species when adsorbed on oxygen sites. In resume, gas-sensing mechanism is based on the adsorption of CO_2_ molecules on the metal oxide semiconductor surface in different sites (metal or oxygen sites) at different temperatures resulting in an increase in surface resistance depending upon the concentration of CO_2_ molecules. Detection of low concentrations of CO_2_ has been desired not only for industrial applications but also for environmental purposes (Yoshioka et al., 1991).

On the other hand, in most of the reported literature, flat surfaces have been chosen for the deposition of nanostructured thin films of the oxide layers, such as glass or alumina (Yoshioka et al., 1991; Patel et al., 2003; Shokry Hassan et al., 2013). Not only porous surfaces such as porous silicon can provide better adhesion and a higher surface area for the deposition of metallic oxides, but also the presence of higher density of nucleation sites (fractal structure) results in the formation of nanostructured metal oxides and enhances the sensing response (Utriainen et al., 1997; Stolyarova and Nemirovsky, 2011; Kumar et al., 2012; Martínez et al., 2016; Karthik et al., 2018). Thus, silicon-based hybrid structures open the possibility for futuristic scalable very large-scale integration (VLSI) applications. In addition, Martínez et al. (2016) investigated the growth kinetics of ZnO deposited over pSi substrates through magnetron sputtering and tested as CO_2_ gas sensors. Karthik et al. (2018) fabricated ZnO or SnO_2_ on macroporous silicon substrates. The metal oxides were deposited through soft chemical route utilizing two different sources of metallic ions for CO_2_ gas sensors. When compared to crystalline substrates, the ZnO (or SnO_2_) over pSi presented higher sensing response and lower response times. Mendoza-Agüero et al. (2014) fabricated electrochemically grown WO_3_ films deposited over macroporous silicon substrates. They studied the effect of annealing temperature in the electrical properties of the hybrid structures and utilized them for ethanol vapors sensing at a concentration up to 1 ppm. In all cases, the gas-sensing capability was enhanced by the utilization of pSi substrates due mainly to their large surface/volume ratio.

With respect to the fabrication of metal oxide/carbon composites, ZnO has been explored due to its versatility in terms of different morphological structures that can be formed and its ease of fabrication [different fabrication routes such as precipitation (Srikanth and Jeevanandam, 2009), mechanochemical (Sánchez Zeferino et al., 2016), hydrothermal (Byrappa et al., 2006), or solvothermal (Lu et al., 2008)]. For the fabrication of hybrid structures, alternative methods such as solution- and solid-state pyrolysis of organic materials or drop casting of hydrothermally synthesized CDs offer low-cost and simple fabrication methods of hybrid structures. The above-mentioned characteristics combined with a high-surface area substrate such as porous silicon could enhance the density of the active sites for the possible increment in the sensing response.

In this work, incorporation of nitrogen-doped carbon dots onto ZnO-coated pSi substrates (NCD–ZnO–pSi hybrid structure) has been demonstrated to enhance the CO_2_ sensitivity of the hybrid solid-state gas sensor. With respect to the pristine sample (without NCDs), the gas-sensing response of the proposed NCD–ZnO–pSi hybrid structure increased by ~24 and ~37% under a CO_2_ concentration of 15 ppm for operating temperatures of 100 and 200°C, respectively, opening the possibility for developing low-cost VLSI compatible solid-state sensors.

## Experimental Procedure

### Porous Silicon Substrate Preparation and ZnO/ZnO-CDs Preparation and Deposition

The macroporous silicon substrate was fabricated by electrochemical etching utilizing a 14–22 Ω cm *p-*type silicon wafer with (100) crystalline orientation. The etching procedure was carried out for 10 min utilizing an electrolyte solution composed of dimethylformamide (DMF) and hydrofluoric acid (HF) mixture with a 33:1 volumetric ratio and a constant current of 10 mA/cm^2^. After etching, the pSi substrate was rinsed with ethanol and dried with pentane.

For the ZnO deposition by precipitation method, 0.25 M of zinc acetate [Zn(CH_2_COOH)_2_∙2H_2_O] and 0.25 M of urea (CH_4_N_2_O) were mixed in deionized water and stirred for 30 min at room temperature. For controlling the pH of the solution, sodium hydroxide (NaOH) was added to the mixture until the pH was 8.0, and then, the solution was heated to 90°C. After getting the desired temperature, pSi substrates were immersed in the solution for 30 s, subsequently rinsed with deionized water, and dried at room temperature. Finally, the obtained sample (denoted as ZnO–pSi sample) was thermally treated at 550°C for 6 h in air. The reaction path for the formation of ZnO nuclei in a basic medium can be described through [Equations 1–3; (Morales Flores et al., 2014; Sánchez Zeferino et al., 2016; Karthik et al., 2018)]:

(1) $$\begin{array}{r} \text{Z}{\text{n}}^{2+}+{2OH}^{-}\to Zn{\left(OH\right)}_{2}↓ \end{array}$$

(2) $$\begin{array}{r} \text{Zn}{\left(\text{OH}\right)}_{2}+{2OH}^{-}\to \;Zn{{\left(OH\right)}_{4}}^{2-} \end{array}$$

(3) $$\begin{array}{r} \text{Zn}{{\left(\text{OH}\right)}_{4}}^{2-}\to ZnO+H_{2}O+2OH^{-} \end{array}$$

The NCDs were prepared by hydrothermal method utilizing citric acid and urea as precursor materials (Bogireddy et al., 2019, 2020). Briefly, 0.20 g of citric acid and 0.38 g of urea were mixed in 50 ml of deionized water. The solution was transferred to a Teflon-lined stainless-steel autoclave and heated at 180°C for 1 h and finally cooled to room temperature to obtain the solution. Synthesized NCDs were centrifuged several times, and the supernatant solution was used to approximate the NCD concentration of ~1.0 ± 0.05 mg/ml. NCD–ZnO–pSi hybrid structures were prepared by cast dropping different amounts of NCD solution (0.1, 1.0, 2.0, 3.0, and 4.0 μl, denoted as NCD–ZnO–pSi-0.1, NCD–ZnO–pSi-1, NCD–ZnO–pSi-2, NCD–ZnO–pSi-3, and NCD–ZnO–pSi-4, respectively) diluted in the same volume of deionized water onto the ZnO–pSi hybrid structure. The covered area for the CO_2_ gas sensing was kept constant for all the samples to ~3.2 mm^2^. The deposited NCDs were dried at 40°C for 15 min and then tested for CO_2_ gas sensing.

### Characterization and Electrical Gas Sensor Preparation and Measurements

The morphological features and the elemental composition of the fabricated ZnO–pSi and NCD–ZnO–pSi hybrid structures were analyzed in a Hitachi SU-5000 field-emission scanning electron microscope (FESEM). For the transmission electron microscopy (TEM) measurements of NCDs, they were deposited by drop casting onto a 100-mesh Cu grid with an amorphous carbon film and analyzed in a JEOL JEM-ARM200F transmission microscope operating at 200 kV. To study the optical properties of the NCDs, ZnO–pSi, and NCD–ZnO–pSi hybrid structures, their fluorescence spectra [photoluminescence (PL)], and the photoluminescence excitation (PLE) spectrum were monitored in a Cary Eclipse PL spectrometer. Cathodoluminescence (CL) spectra of the hybrid structures were recorded in a MonoCL4 system attached to the FESEM (20 kV operating voltage), in the range of 1.75–3.50 eV (350–700 nm). X-ray diffraction (XRD) patterns of ZnO–pSi and NCD–ZnO–pSi hybrid structures were recorded in the range of 10–70° utilizing a Bruker D2 Phaser system with a Cu Kα radiation source (λ = 1.54 Å). The absorbance spectrum of the NCDs in distilled water was recorded with dual-beam Perkin Elmer LAMBDA 950 spectrophotometer in the range of 200–400 nm.

For the CO_2_ gas-sensing capability of the ZnO–pSi and NCD–ZnO–pSi hybrid structures, a homemade glass chamber equipped with a ceramic hot plate (controlled by a 200-W dimmer) was utilized to control the temperature. Through silver contacts, the hybrid structures were connected to a computer-controlled Keithley 2400 source meter for monitoring the changes in the electrical resistance of the ZnO–pSi and NCD–ZnO–pSi hybrid structures in air and in the presence of CO_2_ at two operating temperatures (100 and 200°C). The CO_2_ gas flow was injected to the glass chamber by controlling a manual valve at the desired concentrations (5, 10, and 15 ppm). The sensing percentage (%*S*) of the hybrid structures was determined by the relation %$S=\left(\frac{R_{CO_{2}}-R_{a}}{R_{a}}\right)\times 100\%$, where *R*_*C*_*O*__2__ and *R*_*a*_ are the resistances in the presence of CO_2_ and in air, respectively. For comparison purposes, ZnO was deposited onto crystalline silicon (cSi) substrate following the same procedure described earlier for CO_2_ gas sensing.

## Results and Discussion

**Figure 1:**
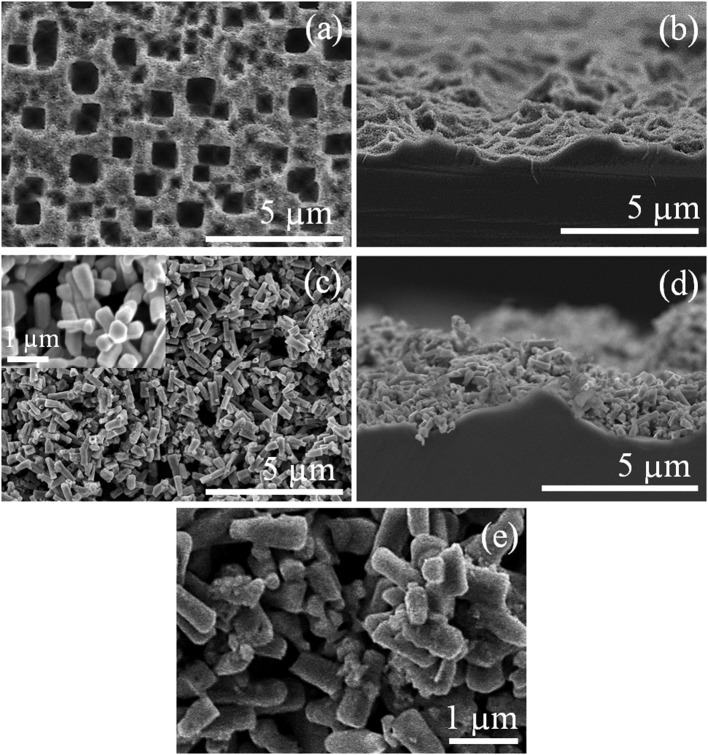
Scanning electron microscopy (SEM) micrographs of the pSi substrate **(a)** before and **(c)** after ZnO deposition (the inset shows an magnified area of the ZnO layer) and **(b**, **d)** their respective cross-section view; **(e)** ZnO–pSi hybrid structure after 1.0 μl nitrogen-doped carbon dots (NCDs) suspension deposition.

The features of the pSi substrate analyzed through SEM before and after the ZnO deposit (Figure 1) reveal square-shaped pores of ~1.0 μm (Figure 1a), and the cross-section of the pSi substrate shows the high roughness of the substrate (Figure 1b), which increases the effective surface area and provides additional nucleation sites for the deposition of ZnO. After ZnO deposition by chemical precipitation (Figures 1c,d), the formation of ZnO micro-/nanorods with a hexagonal cross-section (inset, Figure 1c) on the surface of pSi is observed. The nucleation rate of ZnO is enhanced by the increment in the solution pH, reducing the reaction time needed for the deposition of a micro-/nanostructured layer on the pSi surface, although after the deposition of NCDs by drop casting, one can only observe some randomly distributed clusters of NCDs along the surface of ZnO–pSi (Figure 1e) through scanning electron microscopy. TEM analysis (Figure S1) of NCDs reveals the possible presence of ~3–4-nm-sized NCDs on ZnO nano-/microstructured surface.

**Figure 2:**
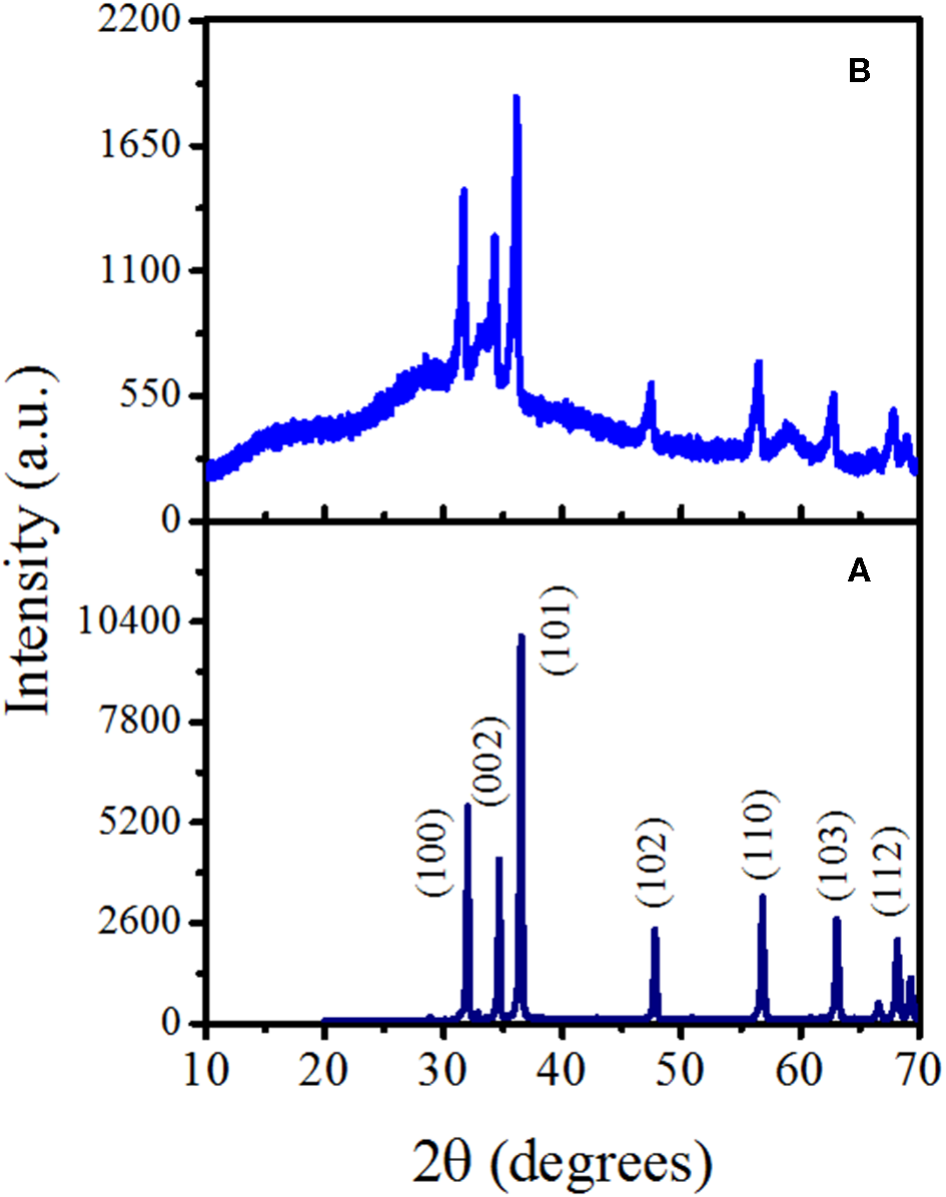
X-ray diffraction (XRD) patterns of the fabricated **(A)** ZnO and **(B)** nitrogen-doped carbon dot (NCD)–ZnO (with 1.0 μl NCDs suspension) hybrid structures.

Figure 2 shows the recorded XRD patterns of the ZnO micro-/nanorods, where their high crystalline quality is noticed. The narrow intense diffraction peaks at 31.7, 34.4, and 36.2° and the low-intensity peaks at 47.5, 56.5, 62.8, and 69.0° can be assigned to the (100), (002), (101), (102), (110), (103), (200), (112), and (201) planes of the wurtzite hexagonal phase [Joint Committee on Powder Diffraction Standards (JCPDS) card no. 79-0207] of the ZnO. The *a* and *c* lattice constants of ZnO were determined with the lattice geometry equation for a hexagonal wurtzite crystalline structure (Equation 4):

(4) $$\begin{array}{r} \frac{1}{d^{2}}=\frac{4}{3}\left(\frac{h^{2}+hk+k^{2}}{a^{2}}\right)+\frac{l^{2}}{c^{2}} \end{array}$$

where *h, k*, and *l* values correspond to the Miller indices of the selected diffraction plane, and *d* is the interplanar distance of such planes, obtained from the XRD patterns according to the Bragg's law (Khorsand Zak et al., 2020). The estimated values for *a* and *c* are 3.23 and 5.17 Å, respectively, which are close to the reported values for ZnO (Kanari et al., 2004; Bitenc et al., 2009; Sánchez Zeferino et al., 2016). The crystallite size (*D*) of the ZnO micro-/nanostructures was estimated utilizing the Debye–Scherrer's formula *D* = *Kλ*/β_*D*_ *cos θ*, where *K* is the shape factor (considered to be 0.9), λ is the X-ray excitation wavelength, β_*D*_ is the full width at half maximum (FWHM) of the selected diffraction peak, and θ is the diffraction angle (Morales Flores et al., 2014; Sánchez Zeferino et al., 2016). The obtained value of *D* for the ZnO micro- /nanostructures is 40.3 nm. On the NCD addition, the diffraction peaks assigned to the NCD–ZnO hybrid structure are less intense and broader than the pristine sample. It is also observed that the diffraction pattern is uplifted compared to the ZnO pristine sample, indicating a reduction in the crystalline quality. Additional wide diffractions signal around 28.5 and 40.7° can be assigned to the presence of the NCDs (Li et al., 2007). Nevertheless, the reported values for different C-based structures lies in the range of 31–36° and 48–53° for the (002) and (100) planes of C, respectively (Zhao et al., 2015). Thus, the angle difference in the XRD pattern of NCD–ZnO hybrid structure could also be by the formation of ternary phases such as zinc carbonate hydroxide on the surface of ZnO micro-/nanostructures (Kanari et al., 2004).

**Figure 3:**
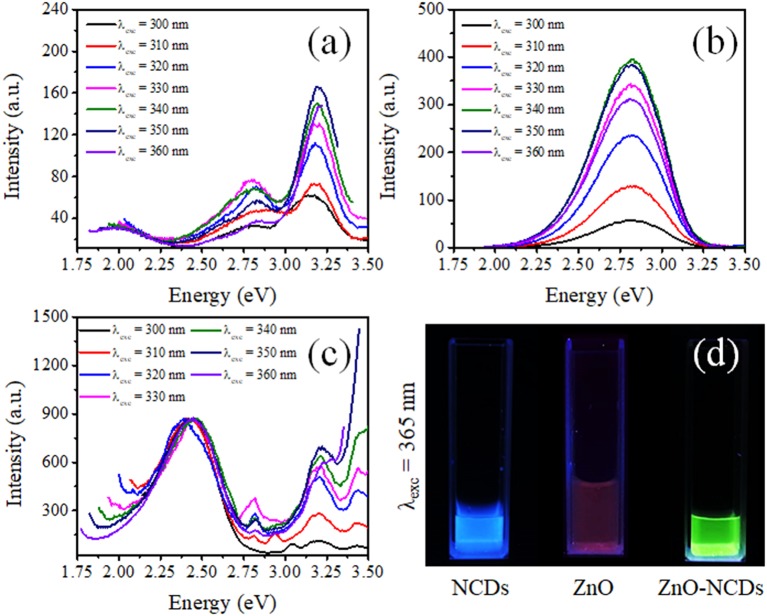
Photoluminescence (PL) spectra of the **(a)** ZnO–pSi pristine structure, **(b)** nitrogen-doped carbon dot (NCD) suspension, and **(c)** NCD–ZnO–pSi hybrid structures with 1.0 μl of NCD suspension (ZnO–pSi and NCD–ZnO–pSi hybrid structures were measured in solid state, and NCDs were measured in aqueous suspension); **(d)** optical image of aqueous suspensions of NCDs, ZnO, and NCD–ZnO illuminated under UV light.

PL spectra of the NCDs (in aqueous suspension), ZnO–pSi, and NCD–ZnO–pSi-1 hybrid structures are shown in Figure 3. For the ZnO–pSi structure, the spectra present two main emissions centered at about 3.23 eV (388 nm) and 2.79 eV (443 nm) and a broad emission centered around 2.00 eV (623 nm) (Figure 3a). On the other hand, the fluorescence spectra of the NCDs with different excitation wavelengths (Figure 3b) presented a well-defined emission band around 2.80 eV (441 nm), with its intensity depending on the excitation wavelength (λ_exc_), which is consistent with its PLE and absorbance spectra (Figure S2) and the reported literature (Zhang et al., 2010; Lim et al., 2015; Bogireddy et al., 2019) (a maximum fluorescence intensity was obtained with λ_exc_ in the range of 340–360 nm). Besides the excitation wavelength, it is well-known that the carbon/graphene dots present emissions in the UV–visible (UV–vis) region depending on their size, shape, and composition (Li et al., 2012). The PL spectra of the NCD–ZnO–pSi-1 structure (Figure 3c) show a very intense broad emission band centered at ~2.46 eV (504 nm) with a reduction in the relative intensity of the bands at 2.0, 2.8, and 3.2 eV observed in the pristine sample. In Figure 3d, the emission of NCDs, ZnO micro-/nanorods, and NCD–ZnO hybrid structure suspensions in deionized water under excitation of a UV lamp (λ_exc_ = 365 nm) are observed.

**Figure 4:**
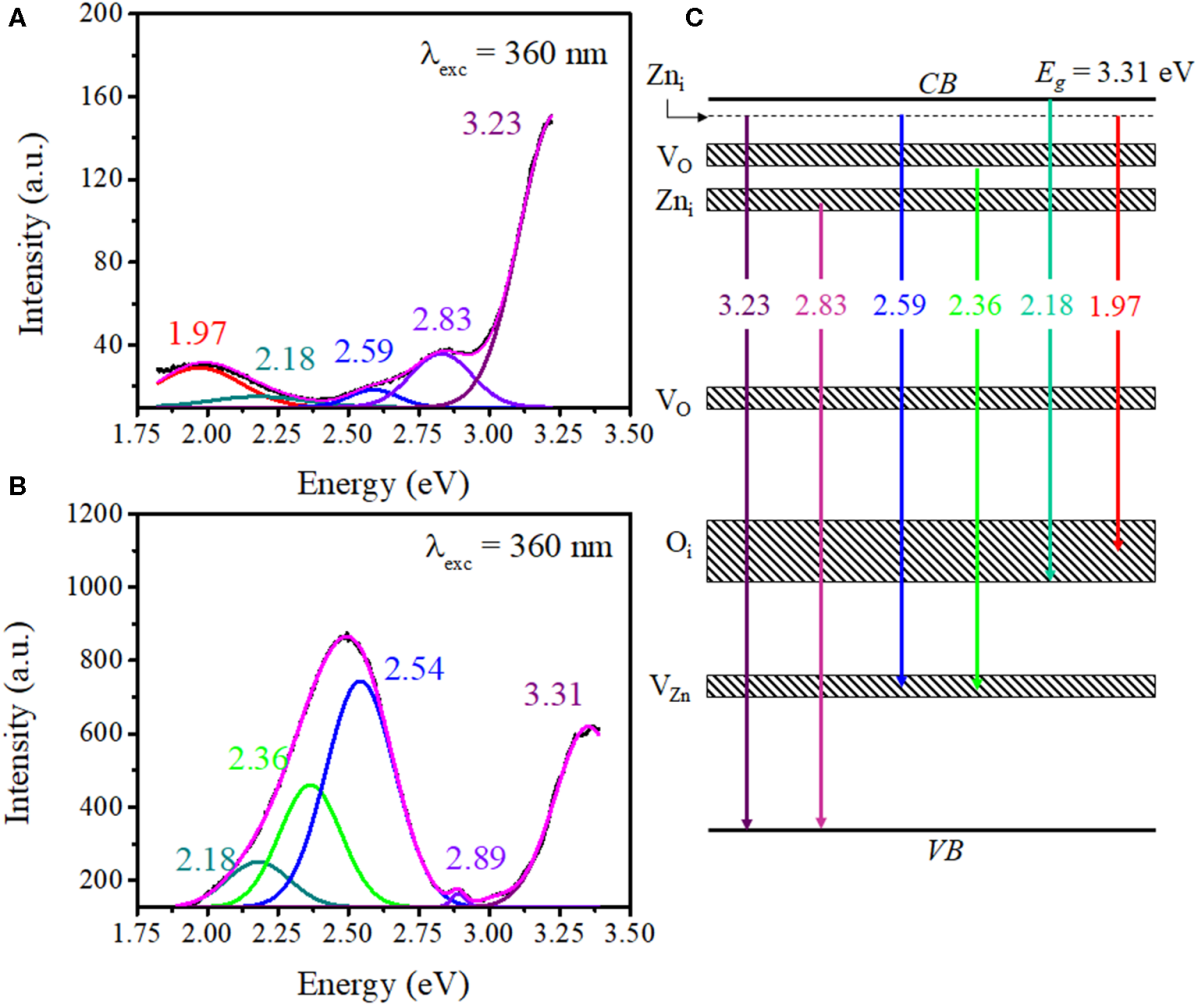
Deconvoluted photoluminescence (PL) spectra of the **(A)** ZnO–pSi and **(B)** nitrogen-doped carbon dot (NCD)–ZnO–pSi hybrid structures under an excitation of 360 nm. **(C)** Proposed band diagram of the transitions in ZnO and NCD–ZnO hybrid structures.

The Gaussian deconvolution of spectra corresponding to ZnO–pSi and NCD–ZnO–pSi-1 hybrid structures revealed the bands attributed to their components (Figure 4). For the pristine sample excited with a λ_exc_ = 360 nm, the PL spectrum presents five emission subbands around 1.97 (red band), 2.18 (yellow band), 2.59 (blue band), 2.83 (violet band), and 3.23 eV (ultraviolet band) with predominant UV emission. The ultraviolet band has been associated with the free exciton recombination in ZnO (Escobedo-Morales and Pal, 2008; Morales Flores et al., 2014), while blue emission can be related to the defect–defect transition between zinc interstitials (Zn_i_) and zinc vacancies (V_Zn_) levels (Escobedo-Morales and Pal, 2008). Although their origin is still controversial, yellow-red emissions have been related to the formation of oxygen interstitials (O_i_) in ZnO (Escobedo-Morales and Pal, 2008; Ahn et al., 2009; Morales Flores et al., 2014), and their presence can be correlated with the thermal treatment conditions during the preparation of the ZnO–pSi hybrid structures. The deconvolution of the NCD–ZnO–pSi-1 PL spectrum (Figure 4B) revealed the presence of one additional emission band at 2.36 (525 nm), which has been widely related to the formation of oxygen vacancies (*V*_O_) in different metallic oxides, including ZnO (Leiter et al., 2003; Escobedo-Morales and Pal, 2008; Ahn et al., 2009; Bitenc et al., 2009; Morales Flores et al., 2014; León Sanchez et al., 2015; Ramos Ramón et al., 2018). As observed in the proposed bands diagram (Figure 4C), the emissions in between 2.18 and 2.54 eV are caused by defect–defect transitions (Escobedo-Morales and Pal, 2008). Although the appearance of the green bands and the red band quenching on the incorporation of NCDs can be caused by the reduction in the O_i_ sites due to the formation of ZnO–carbon functional groups bindings such as carboxyl (–COOH), hydroxyl (–OH), or epoxy through Zn–O–C bonding or by Zn^2+^ ions bonded to NCDs (Son et al., 2012), transitions between the lowest unoccupied molecular orbital (LUMO) from NCDs and the valence band (VB) of ZnO can be overlapped in the photoluminescence signal (Yang et al., 2010; Yin et al., 2011; Son et al., 2012; Lim et al., 2015). As reported by Son et al. (2012), the electrons from the O 2*p* orbital of ZnO are excited to the LUMO levels of NCDs. Then, those photogenerated electrons undergo to their original O 2*p* orbital, giving a green emission observed in the PL spectrum. The presence of NCDs on the surface of ZnO then contributed to the enhancement of green emission, but those NCDs do not present the same emission as in water. While NCDs present high emission in water suspension, it has been reported that their emission intensity depends on the dispersion medium or by its surface passivation and functionalization (Lim et al., 2015). In aqueous medium, the NCDs present a high intense blue emission (as observed in Figure 3d) due to the presence of OH^−^ functional groups of water. Thus, their luminescent effect is quenched by the absence of functional groups on the NCD surface in solid state on the surface of ZnO.

**Figure 5:**
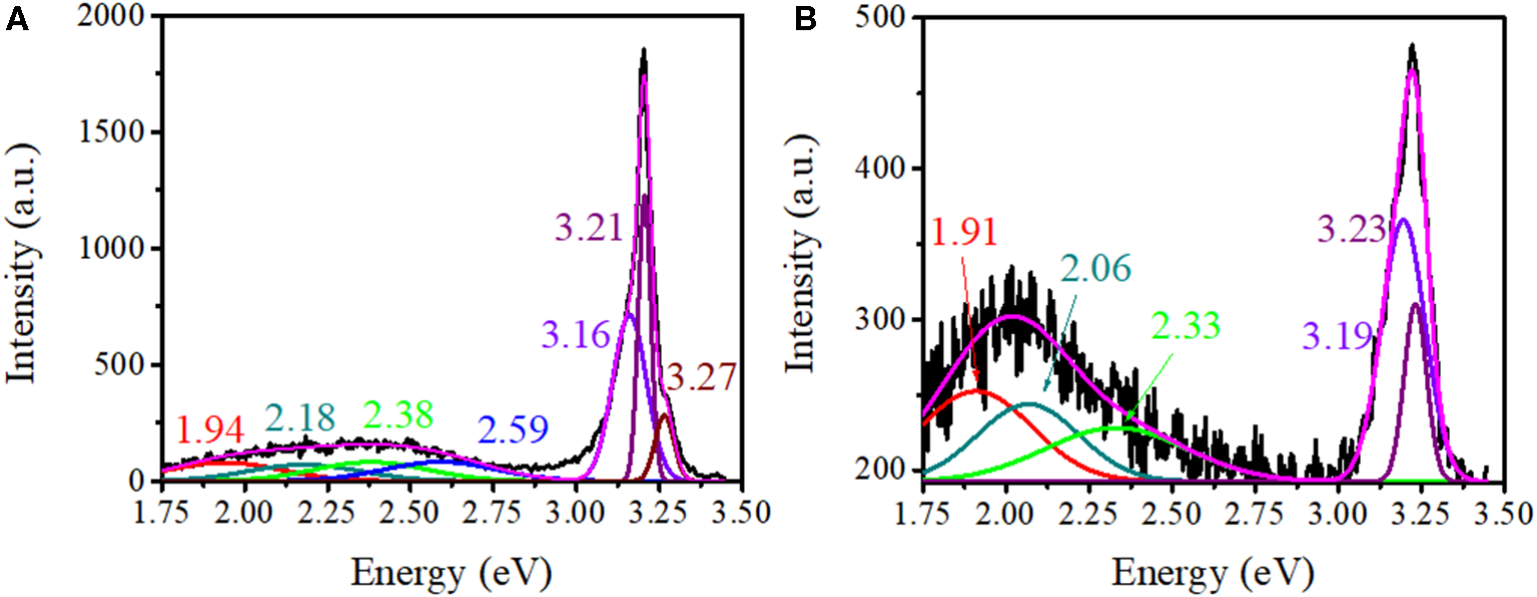
Deconvoluted cathodoluminescence spectra corresponding to **(A)** ZnO–pSi and **(B)** nitrogen-doped carbon dot (NCD)–ZnO–pSi (with 1.0 μl of NCD suspension) hybrid structures.

The intensity of CL is generally a function of the density of the traps/defects in the material (Magdas et al., 2009; Ramos Ramón et al., 2017; Vásquez et al., 2018), and the gas-sensing performance of semiconducting metal oxides is strongly dependent on the microstructure, including compositions, defects, grains, morphology, etc. Hence, in the present study, CL was chosen as one of the characterization technique to analyze the metal oxide (sensing element) before and after the incorporation of NCDs. In addition to XRD analysis, the structural features of ZnO–pSi and NCD–ZnO–pSi hybrid structures are elucidated through room temperature CL spectra (Figure 5). Typically, the analyzed volume in CL spectroscopy measurements results from the electron beam penetration with an excitation energy of 20 kV (normally a few hundreds of nanometers) and the selected area. As the thickness of the ZnO deposited film is ~2 μm (observed in the SEM transverse image; Figure 1d), the obtained luminescence comes exclusively from the ZnO rather than the pSi substrate. The CL spectrum of the ZnO–pSi sample presented an intense emission band centered on 3.20 eV and a broad band with weak emission centered around 2.25 eV (Figure 5). The Gaussian deconvolution of the spectrum revealed the presence of seven component bands centered at 1.94 eV (red band), 2.18 eV (yellow band), 2.38 eV (green band), 2.59 eV (blue band), 3.16 eV (violet band), 3.22 eV, and 3.27 eV (ultraviolet bands). On the other hand, the relative intensity of the oxygen-related emissions (in the range of 1.9–2.3 eV) increased on the incorporation of NCDs, similar to the results observed in the PL analysis. Although the emission band at 1.94 eV has been associated to ZnO defects, the CL emission band has also been previously associated with the thermally activated defects on the grain boundaries of metal oxide microstructures (Magdas et al., 2009). The intensity of the NCD–ZnO–pSi hybrid structure CL spectrum reduced by one order of magnitude compared to the ZnO–pSi spectrum, indicating a reduction in the density of the radiative sites. According to the XRD patterns (refer to Figure 2), the introduction of NCDs reduces the crystalline quality of ZnO, causing the possible formation of non-radiative defects in the NCD–ZnO–pSi hybrid structure. However, these imperfections in the ZnO surface and less crystalline quality enhance the adsorption of atmospheric oxygen, which in turn resulted in higher sensing responses. The presence of electric field induced internal electric field in ZnO structures, causing an electron diffusion toward the ZnO surface along with CL emission quenching (Bang et al., 2004). As mentioned earlier, the presence of electrons on the surface of metal oxides play an important role in gas-sensing characteristics.

**Figure 6:**
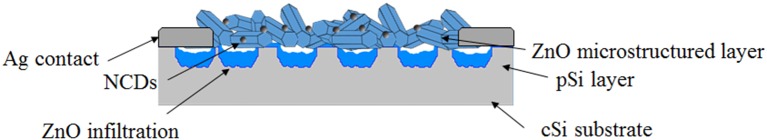
Schematic illustration of the nitrogen-doped carbon dot (NCD)–ZnO–pSi hybrid structure sensor design.

**Figure 7:**
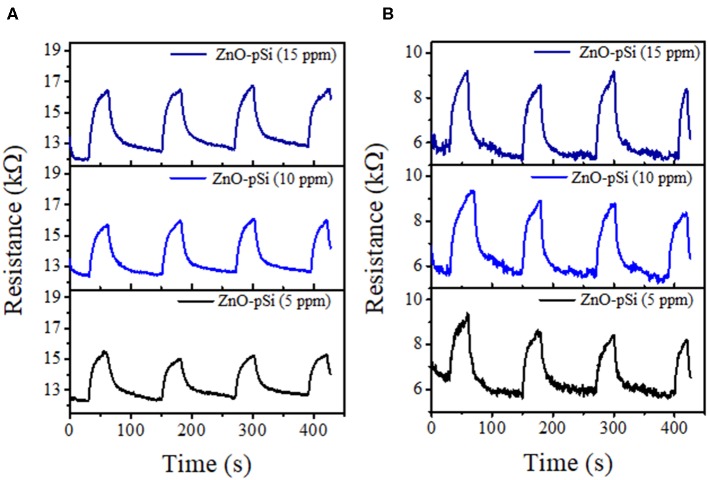
CO_2_ transient response of the ZnO–pSi pristine hybrid structure at **(A)** 100°C and **(B)** 200°C.

To test the applicability of ZnO–pSi hybrid structure as a resistive gas sensor, the sample was put under a CO_2_ flow with three different concentrations and at two different operating temperatures (*T*_S_). The schematic configuration of the fabricated device for CO_2_ sensing is displayed in Figure 6. For the sensing measurements, the sensor resistance was measured without CO_2_ for 30 s for the determination of baseline resistance (*R*_a_), followed by the introduction of CO_2_ for 30 s (ON) for measuring the *R*_*C*_*O*__2__, and finally, the CO_2_ flow was turned off in order to reset the sensor to the baseline status (OFF). After 90 s, the cycle was repeated (Figure 7). For all the measurements, four ON–OFF cycles were carried out in order to confirm the repeatability of the CO_2_-sensing response at *T*_S_ = 100 and 200°C. The resistivity of the hybrid structure increases on the application of gas flow due to the oxidizing characteristic of CO_2_ (Franke et al., 2006).

The hybrid samples fabricated utilizing cSi presented similar behavior when compared to the porous sample for 200°C (Figure S3). The relatively high resistance (higher as compared to pSi substrate) is attributed to poor percolation between the ZnO microstructures formed on cSi. Apart from the formation of hexagonal cylindrical structures, due to the *in situ* formation of ZnO onto the porous silicon fractal substrate, a good adhesion and percolation of ZnO microstructures onto the substrate is assured.

**Figure 8:**
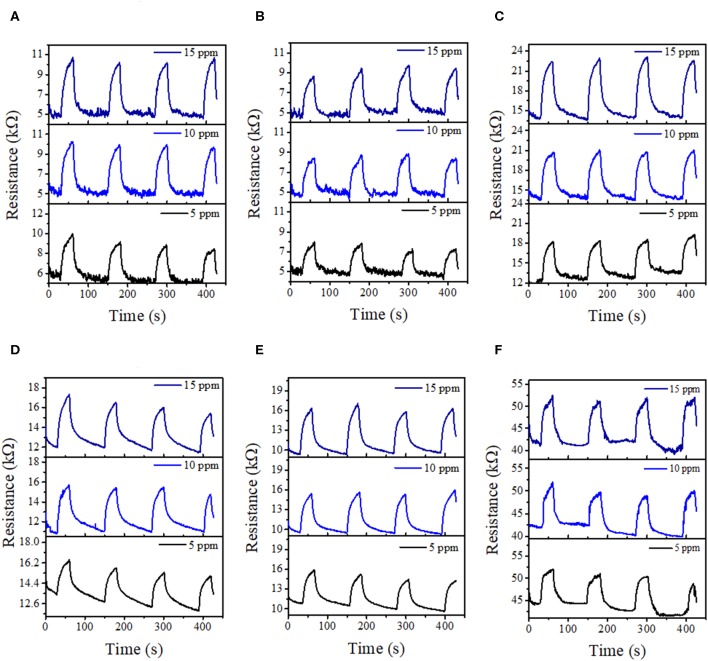
CO_2_ transient response at 100°C (upper row) and 200°C (lower row) of the NCD–ZnO–pSi hybrid structures with different NCDs contents: **(A,D)** 0.1 μl, **(B,E)** 1.0 μl, and **(C,F)** 4.0 μl.

**Figure 9:**
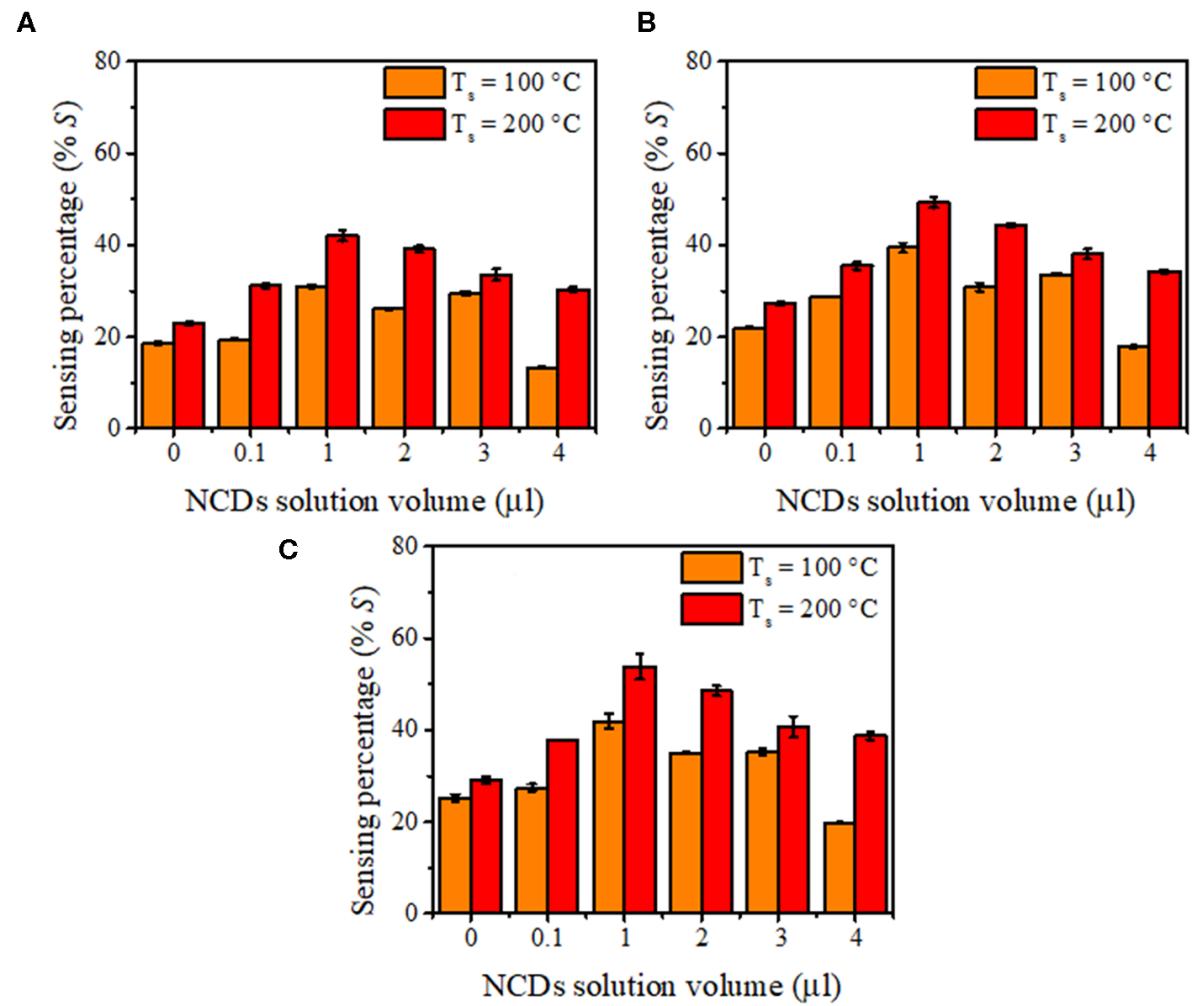
Gas sensing response of nitrogen-doped carbon dot (NCD)–ZnO–pSi hybrid structure when measured with **(A)** 5 ppm, **(B)** 10 ppm, and **(C)** 15 ppm of CO_2_ concentration.

With the addition of NCDs, the sensing response follows similar transient responses as observed for the pristine hybrid structure (Figure 8). The sensing responses of the ZnO–cSi sample results in the sensing response of around 2.9–3.9% at 200°C (Table S1), as compared to one order magnitude higher response from the ZnO–pSi sample (22.9–29.1%) for the same temperature (Figure 9). The increase in the sensing response of ZnO structures over pSi with operating temperature can be correlated to the larger number of reactions between the CO_2_ molecules and the ZnO surface, arising from the high surface porosity and formation of rod-like ZnO structures with higher surface area observed from SEM analysis (refer to Figure 1). On the other hand, while the sensing response of the NCD–ZnO–pSi hybrid structures increased with respect to the pristine porous silicon hybrid structure, reaching a maximum (~42.0–53.9%) for the NCD–ZnO–pSi-1 sample, the sensing response of the NCD–ZnO–cSi hybrid structure reached a maximum of 4.3–6.8% for the cSi substrate. In addition, it is clear from PL and CL measurements (Figures 3–5) that the incorporation of NCDs resulted in the presence of new bands corresponding to the oxygen vacancies, and hence, a higher *V*_O_ density is observed as compared to the pristine hybrid sample. Additionally, from the CL spectrum, a very similar behavior to that of the samples with NCDs is observed, resulting in the presence of yellow and red band, which could be related to the presence of oxygen interstitials. However, any further increase in the NCD content resulted in the gradual decrease in the sensing response (although higher than the pristine control sample), indicating a non-monotonic behavior as a function of NCD content with an optimum of 1.0 μl of NCD solution to maximize the sensing response of the proposed hybrid device (Table 1).

**Table 1:** Sensing response increment on the nitrogen-doped carbon dots (NCDs) addition at two operating temperatures.

**CO_**2**_ concentration**	***T*_**s**_ (^**°**^C)**	**Maximum sensing response increment (%)**
15 ppm	100	24.2
	200	35.5
10 ppm	100	23.9
	200	30.7
5 ppm	100	15.6
	200	29.6

**Figure 10:**
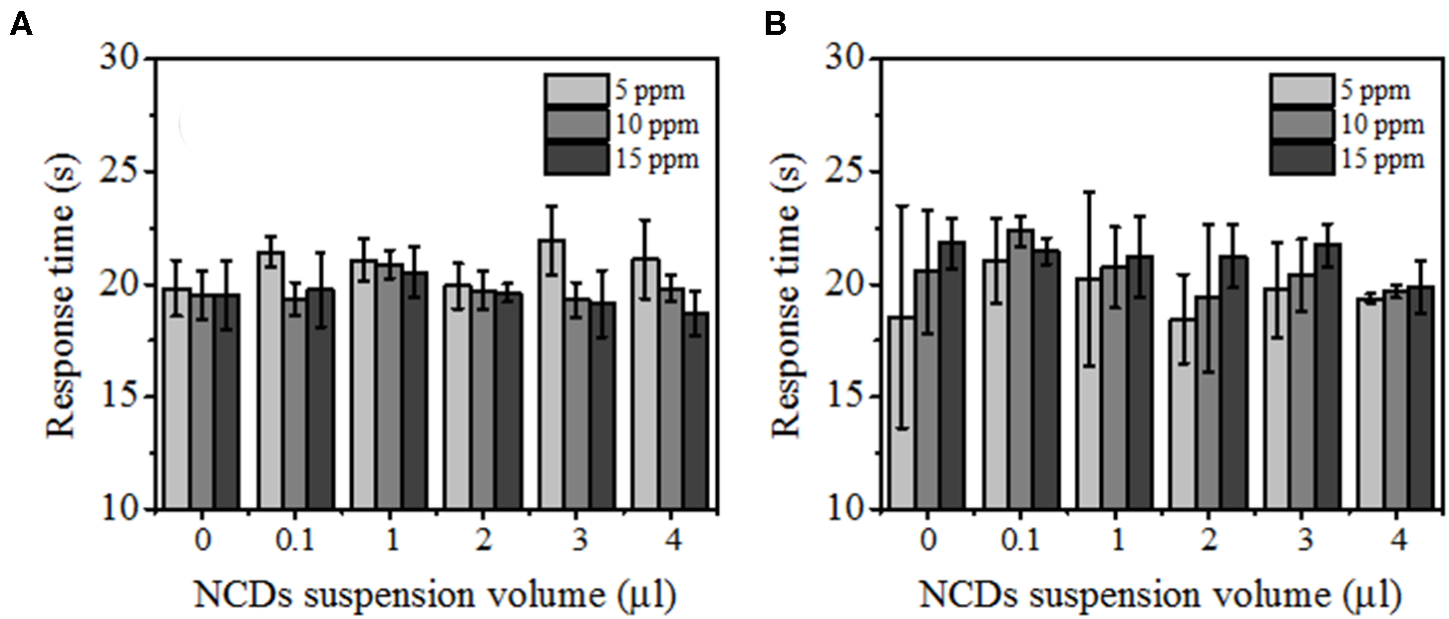
Response time of the fabricated detectors with 0.0, 0.1, 1.0, 2.0, 3.0, and 4.0 μl of nitrogen-doped carbon dot (NCD) solutions under different CO_2_ concentrations with an operational temperature of **(A)** 100°C and **(B)** 200°C.

The response times for the hybrid samples with pSi are summarized in Figure 10. In general, all the proposed sensing samples present a sensing response time of <30 s due to the adsorption–desorption process on the surface of the ZnO–pSi hybrid structure (Varghese et al., 2003; Li et al., 2019). It was observed that, for the measurements carried out at 100°C, the response time slightly decreased on the increment of CO_2_ concentration (Figure 10A), while for the measurements performed at 200°C, the sensing response gradually increased along with the CO_2_ concentration (Figure 10B). On the samples operating at 200°C, the observed opposite behavior has been attributed to the increment in the thermally originated enhanced electron density interacting with the O_2_ molecules present in the chamber, leading to slower CO_2_ adsorption. Since the pristine and NCD-containing hybrid structures have the same measurement parameters, such as operating temperature (either 100 or 200°C), applied current (160 μA), and sensing surface area (~3.2 mm^2^), the resistance values under air atmosphere are inversely proportional to the electron density of the ZnO–pSi and NCD–ZnO–pSi hybrid structures. In Figure S4, the resistance values as a function of the amount of NCD suspension deposited onto the ZnO–pSi hybrid structure are shown, where the resistance values are lower for the NCD–ZnO–pSi-1 and NCD–ZnO–pSi-2 samples, indicating their higher electron density, which is consistent with the observed results.

**Figure 11:**
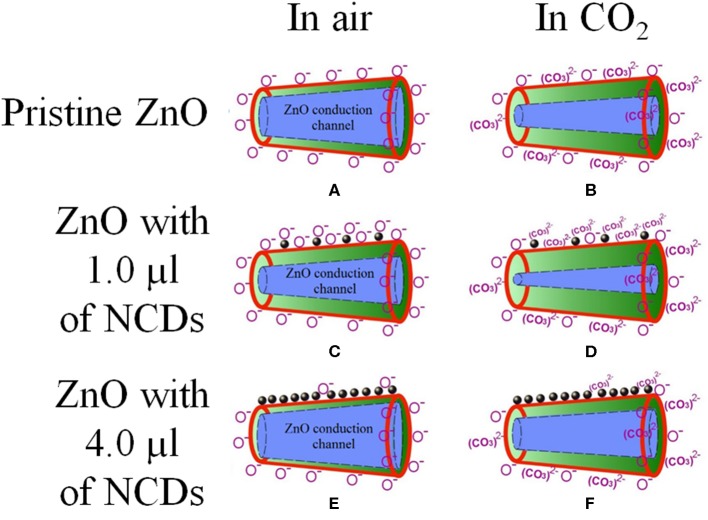
Schematic model of the oxygen adsorption (left column) and their respective CO_2_ reaction with oxygen (right column) of **(A,B)** pristine, **(C,D)** 1.0 μl NCDs, and **(E,F)** 4.0 μl NCDs on the NCD–ZnO–pSi hybrid structure; the NCDs, ZnO surface, and depletion layer are denoted by black-filled circles, red line, and green color, respectively.

It is well-known that the *n*-type characteristic of ZnO (similar to other metal oxides) is caused by the oxygen-related surface defects such as *V*_O_, which act as electron donors (Lin and Jia, 2005; López et al., 2012). At room temperature, free electrons extracted from the *V*_O_ sites are trapped by O_2_ molecules as ionized species (O^−^ or O${}_{2}^{-}$), generating a depletion layer (Schottky potential barrier) (Franke et al., 2006; Li et al., 2019), and hence, the generated potential barrier increases the ZnO resistivity. In addition, the quasi-1-D morphologies of the ZnO micro-/nanorods act as conduction channels (percolation path) with a relatively low electrical resistivity (Figure 11A). However, the grain boundaries between two consecutive micro-/nanorods, their dimensions (length and width), and their random distribution along the film might create additional potential barriers, increasing the electrical resistivity of the micro-/nanostructured ZnO film (Barsan and Weimar, 2001). On the operating temperature increment to *T*_S_, the adsorbed O_2_ molecules are desorbed from the ZnO surface, releasing the trapped electrons and reducing the resistivity. Additionally, thermally generated electrons also contribute to the resistivity reduction. It is well-known that free-electron density in ZnO increases along with the temperature. Hence, the relatively higher sensing response at 200°C (as compared to 100°C) can be explained. With the flow of CO_2_, free electrons are trapped due to electron transfer from ZnO to the adsorbed CO_2_, increasing the ZnO depletion layer and resistivity (Takeuchi et al., 1991). The sensing response also increases along with CO_2_ concentration due to the increment of surface reactions and the formation of ionized (CO_3_)^2−^ species [(Equation 5; (Burghaus, 2014; Karthik et al., 2018); Figure 11B)].

(5) $$\begin{array}{r} \text{C}{\text{O}}_{2}\left(g\right)+O^{-}+e^{-}\to {\left(CO_{3}\right)}^{2-} \end{array}$$

Once the CO_2_ flow is turned off, the CO_2_ molecules are gradually desorbed, reversing the electron transfer and reducing the ZnO resistivity. Furthermore, upon the incorporation of NCDs on to the ZnO–pSi hybrid structure, there is formation of a metal–semiconductor junction, where electrons are transferred from ZnO to NCDs (Li et al., 2019). In addition, it is clear from PL and CL measurements (Figures 3–5) that the incorporation of NCDs resulted in the presence of new bands corresponding to the oxygen vacancies, and the *V*_O_ site density is more compared to the pristine hybrid sample. These *V*_O_ sites increase the conductivity of the hybrid structure, and they adsorb oxygen molecules for capturing electrons from ZnO, enhancing electron exchange and the formation of (CO_3_)^2−^ species (Figure 11C), which is commonly reported as spillover mechanism (Matsushima et al., 1988; Conner and Falconer, 1995; Parambhath et al., 2011; Li et al., 2019). It contains the dissociation of gaseous species on the surface of the catalyst. The dissociated species can diffuse into the host lattice or distribute on the surface of the catalyst, increasing the formation of oxygen ion species by chemical reduction and facilitating the adsorption of oxygen molecules from the atmosphere. When the temperature is increased to the *T*_S_, these O_2_ molecules are desorbed, releasing the trapped electrons and reducing the ZnO electrical resistance. Consequently, the resistance increment is larger due to the formation of a higher active site density, increasing the sensing response of NCD–ZnO–pSi hybrid structures under CO_2_ atmosphere when compared to the ZnO–pSi hybrid structure (Figure 11D). However, with any further increment in carbon content (NCD–ZnO–pSi-2 to NCD–ZnO–pSi-4 samples), the sensing response gradually reduces (Figures 11E,F), indicating the importance of the optimal concentration of NCDs for the required enhancement in the sensitivity.

Some experiments were performed on pristine ZnO–cSi with RH <20 and ~30%, and the results (Table S1) did not reveal any drastic change in the sensing percentage. It has been reported that humidity levels can improve the sensing response of the fabricated gas sensors (Marsal et al., 2003) or affect their performance (Bârsan and Weimar, 2003) depending on different conditions, such as operating range of temperatures, type of gas (oxidizing or reducing), or type of metal oxide (*n*- or *p-*type). In specific cases, the performance of the gas sensors is not significantly affected by the presence of humidity (Lee et al., 2001). As measuring CO_2_ responses in a wide range of conditions (such as gas concentration, temperature, or humidity level) before calibration can lead to the development of an operating model to quantify the gas concentration faster and more accurately, further work with RH quantification is needed.

High surface porosity, low crystalline quality, additional oxygen adsorption sites, and formation of metal-like clusters are some of the main characteristics for obtaining a fast and highly responsive gas sensor. All the above-mentioned parameters have been analyzed by different structural, optical, and morphological characteristics and correlated with gas-sensing properties in this work. Hence, an increment in the CO_2_ gas-sensing response of the ZnO–pSi hybrid structures has been demonstrated using low concentrations of hydrothermally prepared NCDs. The fabricated samples have a competitive CO_2_-sensing response as compared to other reported metal-oxide-based sensors with 100/200°C of operating temperature (Table 2). The short response times (below 25 s) from the synthesized NCD–ZnO–pSi hybrid structure is a major parameter for its potential application as efficient gas sensors. In addition, it has been demonstrated that only the incorporation of optimum concentration of NCDs can result in the enhanced availability of surface-active sites, resulting in maximizing the sensing response of the proposed hybrid structure.

**Table 2:** Comparison of gas-sensing features of metal-oxide-based CO_2_ sensors.

**Material**	**Method**	**CO_**2**_ sensing response**	***T_***s***_* (^**°**^C)**	**Response time (s)**	**References**
SnO_2_ on pSi	Precipitation	19.0	300	~80	Karthik et al., 2018
ZnO on pSi	Precipitation	9.0	300	~65	Karthik et al., 2018
HgSe-ZnO	Wet chemical method	0.2	200	–	Choi et al., 2019
ITO	Evaporation	1.8	200	–	Patel et al., 2003
NCD–ZnO–pSi	Precipitation/drop casting	2.2	200	19	This work

The stability of gas sensors and the quantification of gas concentration have been a matter of concern to the scientific community. Factors like influence of humidity, additional interference of atmospheric gases, non-linear response of conductivity with respect to the gas species, drift with passage of time due to deterioration of the active layer, thermal stress caused by the sensor's heating element, change in the stability of the sensor heater, power consumption, etc., make the quantification of sensing response more challenging (Wang et al., 2010). In general, quantification is performed with an array of sensors in order to eliminate the above effects (Collier-Oxandale et al., 2018; Chegereva et al., 2020). However, Gregis et al. (2018) recently reported the quantification of volatile organic compound (VOC) using single tin oxide gas sensor and utilized the sensors as markers for bioimaging. In their work, authors quantified four different gases utilizing single SnO_2_ sensor based on its surface conductivity change, which made their usage as biomarkers and also can be used in breath analyzers. However, authors could not eliminate the effect of H_2_O and CO_2_ adsorption at concentrations above 40,000 ppm. Hence, in spite of different authors' (Masson et al., 2015; Sauerwald et al., 2018; Lu et al., 2019) attempt to quantify wide range of gases for different industrial applications, it is still challenging due to the effect of heater drift and different atmospheric compositions. It is important to quantify the sensing responses utilizing mathematical models and different calibration strategies based on the sensor device configuration in order to finally utilize the sensors in real-time applications. This quantification results in the usage of devices like indoor air quality measurement, real-time gas monitoring devices in mines detection of hydrocarbons, and as industrial smoke detection devices.

## Conclusions

In summary, ZnO–pSi and NCD–ZnO–pSi hybrid structures were fabricated for the detection of different concentrations of CO_2_ at two different operational temperatures utilizing simple easy chemical synthesis routes. Hydrothermally prepared NCDs were deposited onto the porous substrates covered with ZnO–pSi hybrid structures by drop-casting technique and characterized using FESEM and PL and CL spectroscopies. XRD patterns confirmed the crystalline quality of the hybrid structures. The optical analysis of the fabricated samples, after the incorporation of NCDs on the surface of the ZnO–pSi hybrid structures, revealed a relative increase in the defect-related emissions, which could lead to an increase in active surface sites and hence an increase in the sensitivity. The enhancement in the sensing response is explained through the increment in the free electron density caused by the metal–semiconductor type junction of NCD clusters and ZnO micro-/nanorods and oxygen ion adsorption enhancement associated with the spillover effect. However, a non-monotonic dependence of the sensing response with NCD concentration has been attributed to the reduction in active sites due to a possible decrease in the ZnO surface area available for the CO_2_ adsorption. The fabricated samples demonstrated response times of <25 s, which is a major characteristic of the chemical gas sensors. The sensing response increments of ~24% at 100°C and ~36% at 200°C on NCD-incorporated samples with respect to the ZnO–pSi sample demonstrate their applicability at relatively low temperature (100°C) and CO_2_ concentrations, opening the possibility of developing gas sensors with easy fabrication methods and low-cost materials.

## Data Availability Statement

The datasets generated for this study are available on request to the corresponding author.

## Author Contributions

JR-R and NB prepared the samples. JR-R and JG performed the characterizations and sensing tests. VA planned and coordinated the research. JR-R, TK, and VA analyzed the results. All authors have read and approved the article.

## Conflict of Interest

The authors declare that the research was conducted in the absence of any commercial or financial relationships that could be construed as a potential conflict of interest.
