# Supplementary material for: Nitrogen-Doped Carbon Dots Induced Enhancement in CO2 Sensing Response From ZnO–Porous Silicon Hybrid Structure
Source: Front Chem. 2020 May 5;8:291. doi: 10.3389/fchem.2020.00291 (PMC7214820; doi:10.3389/fchem.2020.00291)
Supplement: Supplementary file 1 [file Presentation_1.pdf]

Supporting information for the manuscript

Nitrogen-doped carbon dots induced enhancement in CO_2 sensing response from ZnO-porous silicon hybrid structure

J. A. Ramos-Ramón[1], N. K. R. Bogyreddy[1], J. A. Giles Vieyra[2], T.V.K. Karthik[3], and V. Agarwal[1*]

[1]Centro de Investigación en Ingeniería y Ciencias Aplicadas, Universidad Autónoma del Estado de Morelos, Morelos, Mexico.
[2]Instituto Tecnológico de Zacatepec, Instituto Nacional de México, Morelos, Mexico.
[3]Ingeniería Industrial, Universidad Autónoma del Estado de Hidalgo, Hidalgo, Mexico.

*** Correspondence:**
V. Agarwal
vagarwal@uaem.mx

High resolution transmission electron microscope (HRTEM) analysis revealed that there are many particles with narrow size distributions (Figure S1). The synthesized nitrogen-doped carbon dots (NCDs) with average size of 3.5 ± 0.5 nm.

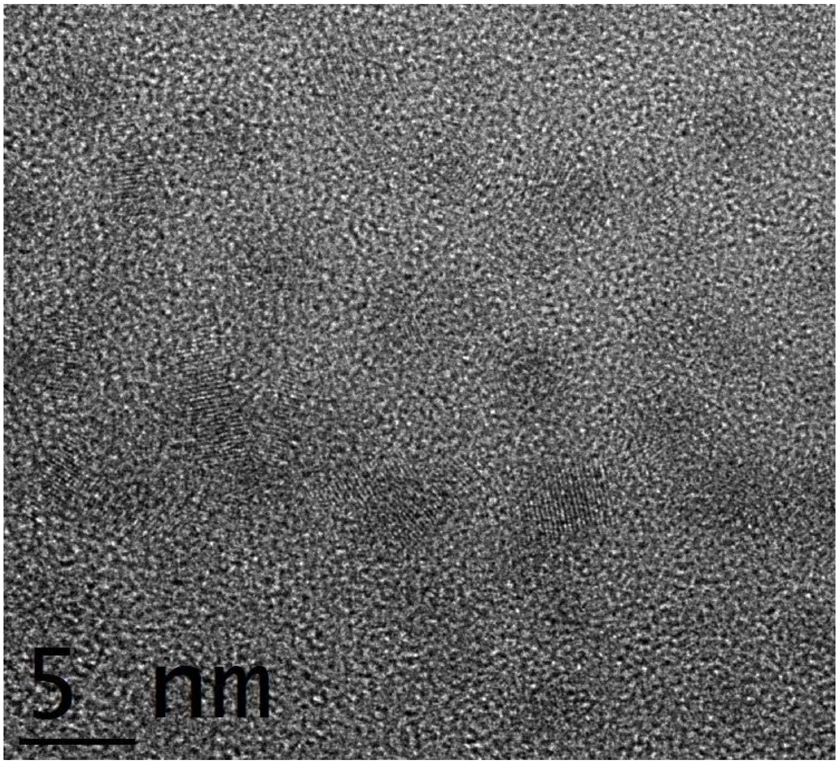

Fig. S1 HRTEM image of the as-prepared NCDs prepared by hydrothermal synthesis route.

The absorbance spectrum of the as-prepared NCDs shows a broad absorption centered at ~340 nm and a pronounced slope around 250 nm (Figure S2 (red curve)). Complementary to the observed in the absorbance spectrum, the PLE response of the NCDs is centered at 344 nm (λ_{em} fixed at 441 nm) (Figure S2 (black curve)).

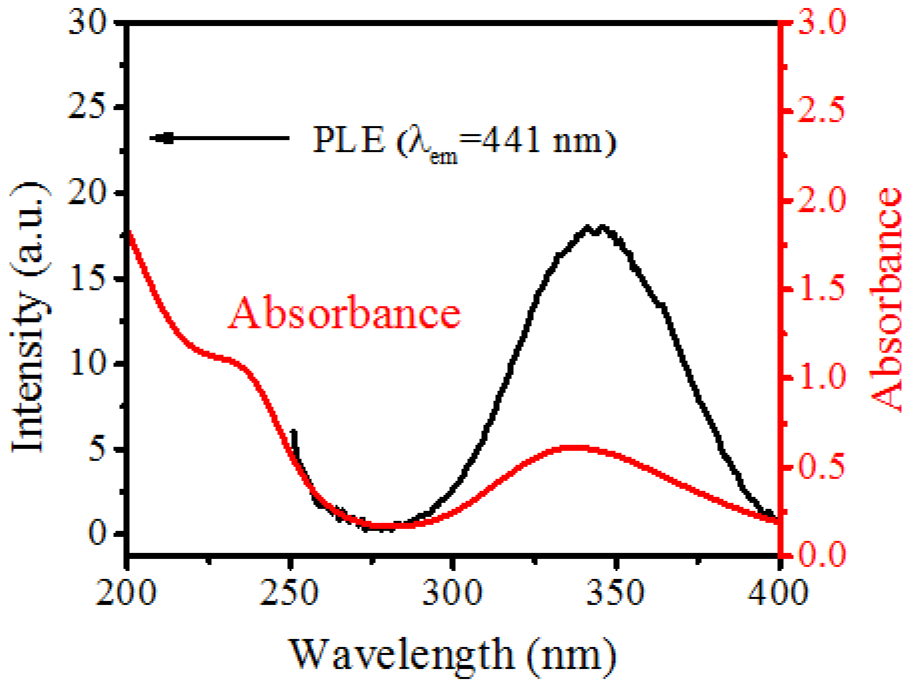

Fig. S2 Photoluminescence excitation (PLE) and UV-Vis absorbance spectra of the NCDs.

In the Fig. S3, the transient response of the reference device fabricated with ZnO on crystalline silicon (cSi) substrate (lower curve) along with two representative NCDs contents (middle and upper curves), where the increment in the sensing response along with the CO_2 concentration in the chamber is observed.

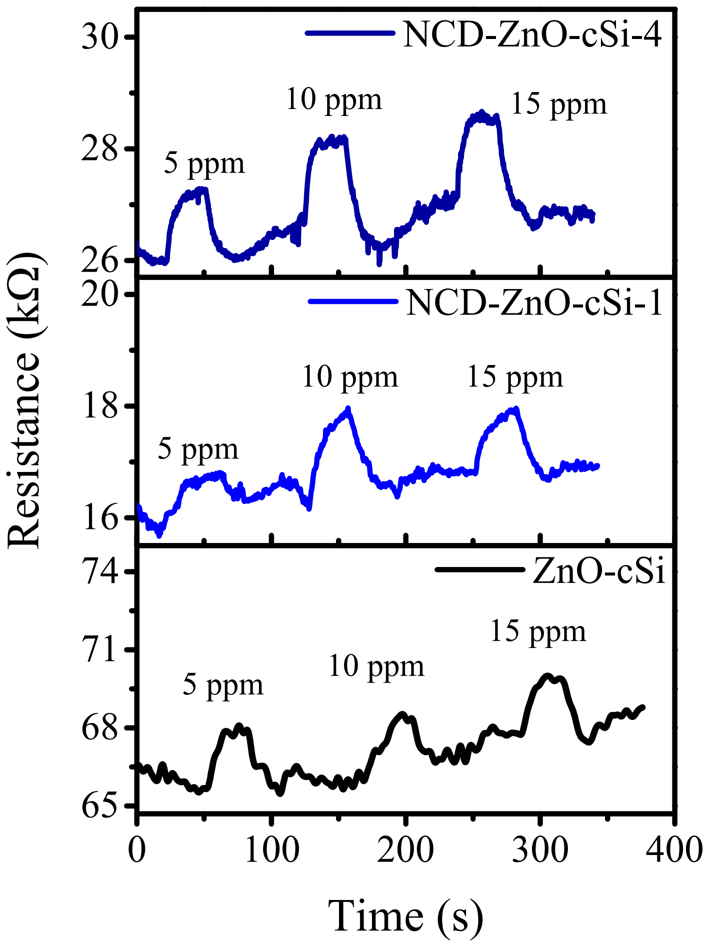

Fig. S3 Representative transient responses of the NCD-ZnO-cSi hybrid structures under different CO_2 concentrations at operating temperature of 200 °C.

Table S1 Sensing response of the NCD-ZnO-cSi hybrid structures with different NCDs content (operating temperature = 200°C).

NCDs solution volume	Sensing percentage (%*S*)		
	5 ppm	10 ppm	15 ppm
0.0 μL	3.0 ± 0.5	3.9 ± 0.5	3.3 ± 0.3
*0.0 μL	3.6 ± 0.9	3.5 ± 1.0	3.2 ± 0.4
1.0 μL	3.6 ± 0.9	1.9 ± 0.4	1.6 ± 0.1
2.0 μL	1.3 ± 0.5	3.6 ± 0.9	4.2 ± 0.3
3.0 μL	4.3 ± 0.4	5.6 ± 0.6	6.8 ± 0.9
4.0 μL	3.9 ± 0.5	5.9 ± 0.6	4.8 ± 0.3

*This sample was measured with a RH ~30%

In the figure S4, the resistance values as function of the amount of NCDs suspension deposited onto the ZnO-pSi hybrid structure are shown. The pristine sample has been represented by the 0 µl value. It can be readily seen that the resistance values are lower for the samples with 1 or 2 µl of NCDs suspension, indicating their higher electron density.

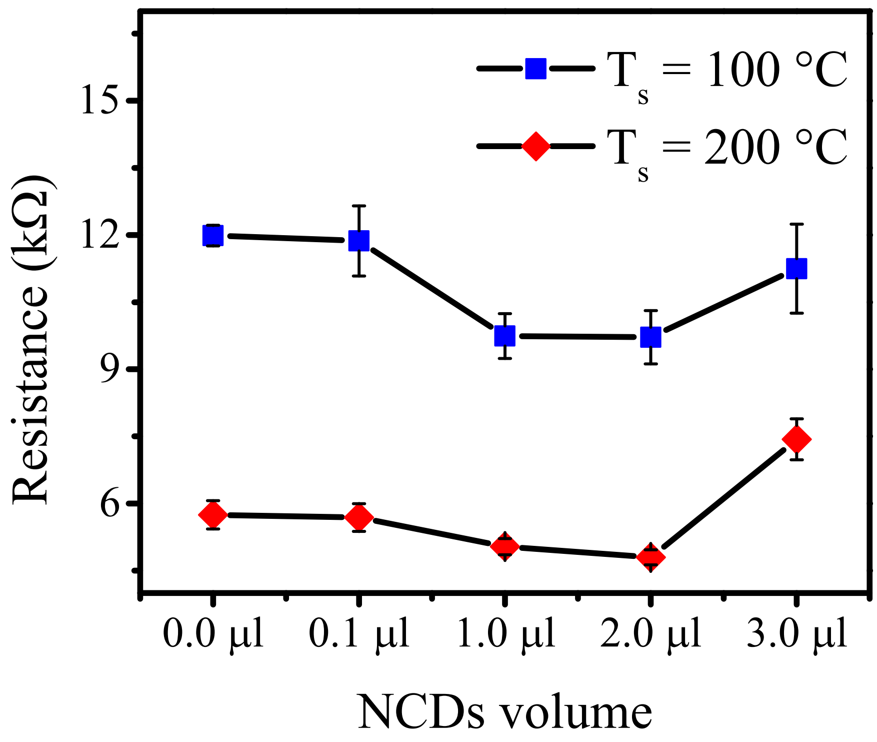

Fig. S4 Electrical resistance of the hybrid structures at the two operating temperatures.
